# The efficacy of antidepressant medication and interpersonal psychotherapy for adult acute-phase depression: study protocol of a systematic review and meta-analysis of individual participant data

**DOI:** 10.1192/bjo.2021.4

**Published:** 2021-02-19

**Authors:** Ellen Driessen, Zachary D. Cohen, Myrna M. Weissman, John C. Markowitz, Erica S. Weitz, Steven D. Hollon, Dillon T. Browne, Paola Rucci, Carolina Corda, Marco Menchetti, R. Michael Bagby, Lena C. Quilty, Michael W. O'Hara, Caron Zlotnick, Teri Pearlstein, Marc B. J. Blom, Mario Altamura, Carlos Gois, Lon S. Schneider, Jos W. R. Twisk, Pim Cuijpers

**Affiliations:** Department of Clinical Psychology, Behavioural Science Institute, Radboud University, the Netherlands; and Depression Expertise Center, Pro Persona Mental Health Care, the Netherlands; Department of Psychiatry, University of California Los Angeles, USA; Columbia University Vagelos College of Physicians & Surgeons, New York State Psychiatric Institute, USA; Columbia University Vagelos College of Physicians & Surgeons, New York State Psychiatric Institute, USA; Department of Psychology, University of Pennsylvania, USA; Department of Psychology, Vanderbilt University, USA; Department of Psychology, University of Waterloo, Canada; Department of Biomedical and Neuromotor Sciences, University of Bologna, Italy; Department of Experimental, Diagnostic and Specialty Medicine, University of Bologna, Italy; Department of Biomedical and Neuromotor Sciences, University of Bologna, Italy; Departments of Psychology and Psychiatry, and Graduate Department of Psychological Clinical Science, University of Toronto Scarborough, Canada; Department of Psychiatry, University of Toronto, Canada; Department of Psychological and Brain Sciences, University of Iowa, USA; Departments of Psychiatry and Human Behavior, Medicine, and Obstetrics and Gynecology, Brown University, USA; and Butler Hospital, USA; Alpert Medical School of Brown University, USA; Parnassia Groep, the Netherlands; Department of Clinical and Experimental Medicine, University of Foggia, Italy; Department of Psychiatry, University of Lisbon, Portugal; Department of Psychiatry and Neuroscience, Keck School of Medicine of USC, USA; Department of Epidemiology and Biostatistics, Amsterdam University Medical Centers, the Netherlands; Department of Clinical, Neuro and Developmental Psychology, Amsterdam Public Health Research Institute, Vrije Universiteit Amsterdam, the Netherlands

**Keywords:** Depression, antidepressant medication, interpersonal psychotherapy, efficacy, individual participant data meta-analysis

## Abstract

**Background:**

Antidepressant medication and interpersonal psychotherapy (IPT) are both recommended interventions in depression treatment guidelines based on literature reviews and meta-analyses. However, ‘conventional’ meta-analyses comparing their efficacy are limited by their reliance on reported study-level information and a narrow focus on depression outcome measures assessed at treatment completion. Individual participant data (IPD) meta-analysis, considered the gold standard in evidence synthesis, can improve the quality of the analyses when compared with conventional meta-analysis.

**Aims:**

We describe the protocol for a systematic review and IPD meta-analysis comparing the efficacy of antidepressants and IPT for adult acute-phase depression across a range of outcome measures, including depressive symptom severity as well as functioning and well-being, at both post-treatment and follow-up (PROSPERO: CRD42020219891).

**Method:**

We will conduct a systematic literature search in PubMed, PsycINFO, Embase and the Cochrane Library to identify randomised clinical trials comparing antidepressants and IPT in the acute-phase treatment of adults with depression. We will invite the authors of these studies to share the participant-level data of their trials. One-stage IPD meta-analyses will be conducted using mixed-effects models to assess treatment effects at post-treatment and follow-up for all outcome measures that are assessed in at least two studies.

**Conclusions:**

This will be the first IPD meta-analysis examining antidepressants versus IPT efficacy. This study has the potential to enhance our knowledge of depression treatment by comparing the short- and long-term effects of two widely used interventions across a range of outcome measures using state-of-the-art statistical techniques.

## Background

As depression is ranked as the single largest contributor to global disability,^1^ there is a great need for effective and efficient depression treatments. Antidepressant medications and different psychological therapies constitute the predominant treatments for depressive disorders.^2^ Concerning psychological treatments, interpersonal psychotherapy (IPT) is a structured, time-limited intervention specifically developed for the treatment of major depression that focuses on current salient relational and interpersonal experiences. Both antidepressant medication and IPT are recommended treatment options in major practice guidelines for depression.^3,4^

When comparing their efficacy in the acute treatment phase, ‘conventional’ meta-analyses have either reported no significant outcome differences or small effect sizes favouring antidepressants in post-treatment depression levels.^5–7^ However, these meta-analyses have focused on treatment acceptability and depression outcomes assessed at treatment completion, whereas other outcome measures (such as quality of life, social functioning, health status) and longer-term effects are also important to consider when comparing depression treatment efficacy. Moreover, conventional meta-analyses, being based on study-level characteristics extracted from publications, are limited by their dependence on the quality of the published data in which treatment effects can be overestimated.^8^ Thus, they can produce biased results.^8,9^

## Individual participant data meta-analysis

Individual participant data (IPD) meta-analysis is an alternative to traditional meta-analysis that examines treatment effects by combining participant-level data from multiple clinical trials. Although it takes more time, effort and resources to conduct an IPD meta-analysis than a conventional meta-analysis, the IPD approach has a number of advantages. It can facilitate standardisation across studies by applying the same analytic approaches for handling missing data and statistical modelling. It allows the verification of results presented in the original studies, and makes it possible to implement novel, more powerful statistical techniques that were not available at the time of the initial publication.^10^ For these reasons, IPD provides the least biased, most reliable means of evidence synthesis and is considered the ‘gold standard’ in meta-analysis.^9^ We, therefore, aim to conduct a systematic review and IPD meta-analysis to examine the efficacy of antidepressants and IPT as compared in randomised clinical trials of acute-phase treatments for adults with depression on a range of outcome measures assessed at post-treatment and follow-up. Here, we describe the protocol for this study.

## Method

### Design

This study is a systematic review and IPD meta-analysis registered in the PROSPERO International prospective register of systematic reviews (PROSPERO: CRD42020219891). Additional important protocol amendments will be updated in this register. This paper accords with the Preferred Reporting Items for Systematic Review and Meta-Analysis Protocols (PRISMA-P) statement.^11^ The 1 September 2019 was the funding date for this study, after which work on the protocol started. Data extraction will start after PROSPERO registration. The study is expected to be completed 31 March 2023. This protocol builds on and extends prior work by our group.^12,13^

### Eligibility criteria

We will include (a) randomised clinical trials (b) comparing antidepressant medication and IPT (c) in the acute-phase treatment of (d) adults with depression.

We will include studies in which antidepressant medication and IPT are directly compared among participants randomly assigned to these treatments. We will consider any type of standard oral antidepressant medication within the therapeutic dose range (such as, selective serotonin reuptake inhibitors (SSRIs), selective serotonin–noradrenaline reuptake inhibitors (SNRIs), tricyclic antidepressants (TCAs) and monoamine oxidase inhibitors (MAOIs)).

An intervention will be considered to be an IPT when it is a psychotherapy based on the manuals developed by Klerman & Weissman for IPT or for the briefer version called interpersonal counselling.^14–18^ We will include IPT in any delivery format (such as individual, group, face to face, telephone or video-conferencing), as long as a clinician delivers the therapy. Unguided bibliotherapy or unguided internet interventions will thus be excluded. The IPT can be delivered in any setting (for example primary care, out-patient and in-patient psychiatric care) and we will place no restrictions on the number of IPT sessions and the duration of follow-up.

We will only include studies of acute-phase depression treatment. Thus, we will exclude, for example, studies that randomised participants to antidepressants and IPT as maintenance treatments after successful acute treatment. Only data from the relevant phases and comparisons of eligible studies will be included. For example, for studies involving augmentation of antidepressants and IPT following non-response to monotherapy, only study data up until the first triage point for augmentation will be included.

Participants must be at least 18 years old. Studies concerning older adult populations will be included as well. Participants will be considered to have depression if they meet specified criteria (for example DSM, ICD-10) for major depressive disorder or another unipolar mood disorder assessed by means of a semi-structured interview or clinicians’ assessment, or if they present an elevated score above the ‘no depression’ cut-off on an evaluator-assessed, clinician-assessed or self-reported measure of depression. Comorbid mental and somatic disorders will be allowed.

Participant criteria will be assessed at study level. Thus, eligible participants from a study including a wider population (for example adults from a study including both adolescents and adults) will not be included, because the integrity of randomisation within the subgroup of eligible participants could be compromised. No restrictions will be placed concerning the years when the study was conducted, or with regard to publication language, date or status.

### Information sources, search strategy and selection process

To identify studies, we will search a database of randomised clinical trials examining the efficacy and effectiveness of psychological treatments for depression that has been used in a series of published meta-analyses (www.metapsy.org). This METAPSY database was developed through comprehensive literature searches in the bibliographic databases PubMed, PsycINFO, Embase and the Cochrane Library and is updated annually. The search strings use a combination of index terms and free-text words indicative of depression and psychotherapies, with filters for randomised clinical trials. The exact terms for the searches are available from https://osf.io/nv3ea/. Searches will be performed until 1 January 2020.

Two experienced depression treatment researchers will independently screen all records, assess full-text papers for METAPSY database eligibility, and rate the treatment comparison(s) examined. Next, two other experienced depression treatment researchers will independently assess all full-text papers of studies marked as comparing a psychotherapy monotreatment condition against another active monotreatment condition for meeting the inclusion criteria for this work. Disagreements will be resolved through consensus in each phase.

In addition, we will check the references of IPT^5–7,19^ and of psychotherapy versus pharmacotherapy^20^ efficacy and effectiveness reviews, as well as the reference lists of the included studies. We will contact a listserv of members of the International Society of Interpersonal Psychotherapy to request ongoing or unpublished studies, and studies that were missed.

### Data collection

We will contact authors of the included studies and invite them to contribute the participant-level data of their studies. Researchers who share their data will be offered co-authorship for all publications that are based on their study data, inasmuch as they meet internationally accepted criteria for authorship of scientific publications (www.icmje.org). In addition, the collected data will be made available to investigators who contribute data to examine other research questions in the combined data-set. This strategy, used in previous IPD meta-analyses concerning depression treatments,^12,21^ has successfully convinced researchers to share their data.

Contact details of all corresponding authors will be collected from the relevant publications, or if not reported there, through internet searches or personal contacts with other researchers. Corresponding authors will be contacted by email with an invitation outlining the project's goals and asking if they would be willing to collaborate by sharing their participant-level trial data. If the corresponding author does not respond after 3 weeks, a second and third email will be sent. If these attempts fail, the other authors will be contacted in the same way, in this order: first, last, second, third, fourth, etc. In case of non-response to email, a letter will be mailed to the corresponding author (again with three attempts). If still no response is received, we will try to contact the corresponding author by telephone. If the corresponding author does not respond, we will contact the other authors by letter and telephone. If none of the authors respond to these efforts, we shall seek other ways of contacting one of the authors (for example via colleagues who might know them).

Study data will be considered unavailable only if all these attempts fail, or if an author indicates that the participant-level data have not been retained or declines to share these data. This strategy has also been used in a previous IPD meta-analysis of depression treatment^12^ and has proven to be successful in reaching authors.

If the author is willing and able to share their trial IPD, the author will transfer the participant-level data-set, including all outcome variables assessed during and after treatment, both in the antidepressant medication and the IPT condition of the study. Data-sets will be anonymised by the authors prior to transfer, so that no personal data are transferred.

### Data integrity check

After the data-set has been transferred, the file will be checked to examine whether the data received match the data reported in the publication, following Driessen and colleagues.^12^ For both the antidepressant medication and the IPT condition, sample size, number of female participants, mean age, observed mean pre-treatment depression scores, observed mean post-treatment score for the primary depression outcome and the number of missing cases for the latter will be calculated from the data-set received and checked against the published article for this purpose. Discrepancies will be resolved with the authors. In addition, the data will be checked for invalid, out-of-range or inconsistent items.

For each study, we will list all outcome measures and assessment points. From the published articles, we will extract multiple study-level characteristics (country, recruitment method, target group, depression inclusion criteria) and treatment characteristics (antidepressant type, number of IPT sessions, IPT format).

To ascertain antidepressant treatment quality, studies will be assessed for the use of a therapeutic dosage and titration schedule (i.e. therapeutic dose achieved within 3 weeks). Study pharmacotherapy will be deemed adequate if both criteria are met. To ascertain IPT quality, studies will be assessed with respect to using a treatment manual, provision of therapy by specially trained therapists and verification of treatment integrity.^22,23^

Two raters will independently assess the Cochrane risk-of-bias tool for randomised trials at outcome level.^24^ Disagreements will be resolved by consensus. When publications omit reporting relevant information, we will ask the authors. For studies for which IPD were not obtained, we will extract effect-size data at baseline, post-treatment and follow-up for each outcome measure reported.

After checking the data, the data-sets will be standardised.^12^ For this purpose, a copy of each trial's raw data file will be recoded into a data file that matches the IPD meta-analysis database in terms of variables. Next, the individual study data files will be merged into a single database in which each participant is identified by a study ID and a unique individual participant ID. After all data files have been recoded and entered, the data for each study will be checked with the original data file for accuracy.

### Measures

The primary outcome for this study is treatment efficacy as assessed by a continuous measure of depression symptom severity at post-treatment. We have chosen depressive symptoms at post-treatment as the main outcome for this study as we consider this the primary target of antidepressant and IPT treatment. For each trial, we will identify the primary continuous measure of depressive symptom severity defined by the study authors. All instruments explicitly measuring depression qualify in this regard. To account for the likelihood that different primary depression measures will have been used, we will standardise depression outcomes by converting depression scores into *z*-scores within each study^12^ as well as by exploring novel approaches based on equipercentile linking^25^ and item-response theory.^26^ Sensitivity analyses will be conducted using unstandardised scores for each specific measure that is assessed in two or more studies included in the meta-analysis (for example the Hamilton Rating Scale for Depression,^27^ the Beck Depression Inventory^28^).^12^ Sensitivity analyses will also be conducted for studies that include outcomes within specific time windows (for example 12–16 weeks).

Secondary outcomes for this study are depression outcomes at follow-up as well as all outcome measures other than depression symptom severity that are assessed at post-treatment or follow-up in at least two of the included studies. These include quality of life, social functioning and health status. As individual studies will likely assess these domains differently, they will be standardised by converting scores into *z*-scores within each study. The sensitivity analyses described previously for the primary outcome will also be conducted for the secondary outcomes.

### Data analysis

Following Driessen et al,^29^ all data-sets received that contain IPD and at least one relevant outcome measure will be considered for quantitative synthesis. We will conduct IPD meta-analyses according to the one-stage approach, because that provides a more exact likelihood estimate in the case of small studies.^30^ We will conduct IPD meta-analyses using mixed models with restricted maximum likelihood to estimate between-study heterogeneity, which is recommended when there are few studies in the meta-analysis or studies have small sample sizes.^31^ Analyses will be based on intention-to-treat samples including all randomised participants. Follow-up data will be excluded from post-treatment analyses, because additional treatment cannot be controlled. We will assess heterogeneity with the *I*^2^ statistic, which describes the variance between studies as a proportion of the total variance. Mixed-model analyses will be conducted with MLwiN (version 2.35 for PC, Centre for Multilevel Modelling, University of Bristol).

We shall start with a basic model including a main effect for time and a time × treatment interaction.^29^ Twisk and colleagues recommend this approach,^32^ because it adequately accounts for baseline values and has favourable properties concerning missing data, allowing participants with only a baseline value but missing post-treatment and/or follow-up assessments to remain included in the analyses. Time will be treated as a categorical variable to facilitate treatment comparison at the different time points.

To account for clustering of participants within studies, we will estimate a random intercept with respect to the study. We will also estimate a random intercept with respect to participants, and fixed slopes. If adding a random slope for the time × treatment interaction on study level results in a model improvement, models including this random slope will be used for effect estimation.

Using this approach, treatment effects can be directly obtained from the regression coefficient of the time × treatment interaction.^29,32^ The regression coefficients of the time × treatment interactions at post-treatment and follow-up represent the treatment comparisons at these assessment moments. These can be interpreted as Cohen's *d* effect sizes for analyses with *z*-scores as outcome measure, and as mean differences for analyses with unstandardised scores as outcome measure.

A number of sensitivity analyses will be conducted to examine the robustness of the findings. Because outcomes of IPT for depression have been found to be associated with the number of sessions,^7^ we will repeat the analyses in subgroups of studies examining IPT and the briefer interpersonal counselling. Because there are indications that psychological treatments of dysthymia are less effective than psychological treatments of major depressive disorder,^33^ we will repeat the analyses in subgroups of studies that include participants meeting diagnostic criteria for dysthymia versus studies that include participants meeting diagnostic criteria for other unipolar mood disorders.

To examine potential effects of study setting, depression inclusion criteria, age and antidepressant type, we will also repeat the analyses in the following subgroups of studies: primary versus secondary or tertiary care, studies that enrolled participants meeting diagnostic criteria for depression versus studies that enrolled participants presenting an elevated score on a standardised measure of depression, studies that enrolled adult versus studies that enrolled older adult populations, and for subgroups of studies examining different classes of antidepressants (such as SSRI, SNRI, TCA, MAOI).

We will also conduct sensitivity analyses to control for the effects of additional non-study treatment during the trial and in the follow-up period by adding collected data in this regard as covariates to the mixed-effects models. To examine the impact of risk of bias, we will conduct analyses in which we add risk-of-bias items as covariates to the mixed-effects models.^34^ In addition, we will conduct sensitivity analyses only including those studies that score negative on all risk-of-bias criteria assessed. We will examine the impact of treatment quality in the same way.

Furthermore, we will examine possible data-availability bias following prior work.^12^ We will conduct *t*-tests and χ^2^ analyses, comparing the included studies with studies for which no participant-level data were obtained with regard to the extracted study characteristics using SPSS Statistics (version 25 for PC). For studies for which IPD were not obtained, we will calculate Cohen's *d* effect sizes for each relevant outcome measure based on data extracted from the publications. Conventional meta-analysis techniques will be utilised to examine differences in effect sizes between studies that contributed data and studies that did not. More specifically, we will conduct subgroup analyses comparing studies for which IPD were versus studies for which IPD were not obtained using Comprehensive Meta-analysis (version 3.3.070 for PC, Biostat). We will apply a fully random-effects analysis and pool study-to-study variance across subgroups, as is recommended when subgroups involve small numbers of studies.^35^ We will assess potential publication bias by assessing asymmetry in funnel plots for meta-analyses including ten or more trials (regardless of whether or not IPD were obtained), as is recommended by Sterne and colleagues.^36^ We will assess the strength of the body of evidence for each outcome measure based on the number of included studies and participants as well as the quality of the included studies.

## Discussion

We have described the study protocol of a systematic review and IPD meta-analysis examining the efficacy of antidepressant medication versus IPT for adult acute-phase depression. The study aim is to collect the participant-level data of randomised clinical trials comparing these two effective antidepressant treatments and to combine these data in order to conduct IPD meta-analyses on a broad range of outcome measures assessed at both post-treatment and follow-up.

### Strengths and limitations

IPD meta-analysis has a number of advantages over conventional meta-analysis.^12^ Collecting the studies’ primary data provides access to outcome variables that might not have been reported in the published articles. Other advantages include facilitating standardisation across studies by allowing the same analytic approaches to be used for handling missing data and statistical modelling, allowing verification of results presented in the original studies, and making it possible to implement novel, more powerful statistical techniques than were available at the time of publication of older studies.^10^

IPD meta-analyses involve collection of original data from all the relevant trials worldwide. These data require preparation and checking before being included in the meta-analysis, and sometimes demand complex decisions on the data to ensure the accuracy of outcomes. It therefore takes more time, effort and resources to conduct an IPD meta-analysis than a conventional meta-analysis based solely on results extracted from published trial reports.^10^ However, the IPD approach can improve the quality of both the data and the analyses, and thus the reliability of the results. Therefore, it is considered the ‘gold standard’ of meta-analysis.^9^

IPD meta-analyses also have limitations.^12^ First, although IPD meta-analyses are generally considered the most reliable approach to evidence synthesis,^9^ this does not mean that they are bias-free: reviewer selection bias, publication bias and data-availability bias require consideration.^37^ We will attempt to reduce such bias by conducting a systematic literature search, posing no restrictions concerning publication language, date and status, and by empirically examining publication bias as well as data-availability bias. Second, IPD meta-analyses rely on variables previously assessed in individual studies and available across multiple trials. For this reason, it is possible that not all outcome variables of interest can be examined. It is also possible that meta-analyses of different outcomes will be based in different subgroups of studies. Third, it will be necessary to standardise outcome variables in order to conduct meta-analyses including all relevant studies, and doing so can change the original variables’ properties. We aim to overcome this limitation by conducting sensitivity analyses using unstandardised scores. Fourth, although we will combine data from multiple clinical trials with different inclusion criteria, target populations, settings and antidepressant types, generalisability of the findings might still be limited to individuals who volunteer to participate in randomised clinical studies.

### Clinical and scientific relevance

Although IPD meta-analyses have now been used to compare the acute treatment efficacy of antidepressant medication with cognitive–behavioural therapy,^21^ with the cognitive–behavioural analysis system of psychotherapy (CBASP),^38^ as well as with combined treatment of antidepressants and CBASP,^38^ and with combined treatment of antidepressants and psychodynamic therapy,^29^ to date no IPD meta-analysis has been conducted examining the efficacy of IPT. With its focus on current salient relational and interpersonal experiences, IPT provides an important alternative to these interventions and it is included as a treatment of choice in major practice guidelines for depression.^3,4^ Therefore, we believe this study has the potential to contribute to our understanding of treatment for depression by comparing the efficacy of two frequently applied therapies in both the short and longer term on a range of outcome measures across studies using state-of-the-art statistical techniques.

## Data Availability

Data availability is not applicable to this article as no new data were created or analysed for this study protocol paper.
